# Immunopathogenic mechanisms and immunoregulatory therapies in MASLD

**DOI:** 10.1038/s41423-025-01307-5

**Published:** 2025-06-10

**Authors:** Yong He, Yingfen Chen, Shengying Qian, Schalk van Der Merwe, Debanjan Dhar, David A. Brenner, Frank Tacke

**Affiliations:** 1 State Key Laboratory of Drug Research, Shanghai Institute of Materia Medica (SIMM), Chinese Academy of Sciences, Shanghai, China; 2 University of Chinese Academy of Sciences, Beijing, China; 3 Laboratory of Hepatology, CHROMETA Department, KU Leuven, Leuven, Belgium; 4 Department of Gastroenterology and Hepatology, UZ Leuven, Leuven, Belgium; 5 Department of Medicine, School of Medicine, University of California, San Diego, CA USA; 6 Center for Metabolic and Liver Diseases, Cancer Genome and Epigenetics Program, Sanford Burnham Prebys Medical Discovery Institute, La Jolla, CA USA; 7 Department of Hepatology & Gastroenterology, Charité-Universitätsmedizin Berlin, Campus Virchow-Klinikum and Campus Charité Mitte, Berlin, Germany

**Keywords:** MASLD, MASH, inflammation, pathogenesis., Immunology, Mechanisms of disease

## Abstract

Metabolic dysfunction-associated steatotic liver disease (MASLD), previously known as nonalcoholic fatty liver disease (NAFLD), is the most prevalent chronic liver disease worldwide, with an estimated global prevalence of approximately 30%; however, effective pharmacotherapies are still limited due to its complex pathogenesis and etiology. Therefore, a more thorough understanding of disease pathogenesis is urgently needed. An increasing number of studies suggest that MASLD and its progressive form, metabolic dysfunction-associated steatohepatitis (MASH), are driven by chronic overnutrition, multiple genetic susceptibility factors, and pathogenic consequences, including hepatocyte damage and liver inflammation. Hepatic inflammation is the key event fueling the conversion from simple steatosis to steatohepatitis and fibrosis. Current therapies for MASH, including the recently approved thyroid hormone receptor-beta agonist resmetirom or the available incretin mimetics, mainly target metabolic injury to the liver but not inflammation directly. In this review, we provide an in-depth discussion of current data related to the immunological mechanisms of MASLD and summarize the effects of current and experimental therapies on immunoregulation in MASLD.

## Introduction

Metabolic dysfunction-associated steatotic liver disease (MASLD), formerly named nonalcoholic fatty liver disease (NAFLD), affects approximately 30% of the global population, leading to a major health and economic burden [1]. MASLD represents a spectrum of disorders ranging from simple steatosis to metabolic dysfunction-associated steatohepatitis (MASH), ultimately resulting in advanced fibrosis, cirrhosis, and hepatocellular carcinoma (HCC) [2]. It is well established that being overweight or obese is closely related to hepatic steatosis, hepatocyte damage, liver inflammation and fibrosis, which was formally recognized by the term “nonalcoholic fatty liver disease (NAFLD)” in 1980 by Jurgen Ludwig and colleagues [3]. However, the term “nonalcoholic” does not accurately capture the etiology of the disease, alongside the absence of defined clinical “positive criteria” to diagnose this disease. Therefore, an international panel of experts has detailed the rationale for an update of the nomenclature, and metabolic dysfunction-associated fatty liver disease (MAFLD) has been proposed as a more appropriate term to describe this liver disease [4–6]. Notably, continued use of the term “fatty” has been considered to be stigmatizing, restricting the population to those with 2 metabolic risk factors. These concerns led to a multistakeholder effort to develop a consensus on a change in nomenclature and the diagnostic criteria for the condition [7, 8]. Central to this change is the shift from “fatty liver disease” to “steatotic liver disease”, which includes all conditions characterized by abnormal hepatic lipid accumulation and encompasses MASLD, alcohol-associated liver disease (ALD), metabolic dysfunction, alcohol-associated liver disease (MetALD) and other rare causes of liver steatosis [9]. Patients presenting with cardiometabolic risk factors such as obesity, insulin resistance, diabetes, dyslipidemia and alcohol intake below 20 g/day for women or 30 g/day for men are now diagnosed with MASLD [10]. ALD represents a separate group of patients in which > 50 g/60 g of alcohol is consumed daily in females and males [11]. Of particular importance is the new category of individuals with MetALD, whose diagnosis of hepatic steatosis requires the presence of cardiometabolic risk factors (CMRFs) and daily alcohol intake between 20–50 g/day for women and between 30–60 g/day for men [11, 12]. This new nomenclature embraces a more comprehensive and nuanced approach to classifying liver diseases and opens up new avenues for various medical fields, including primary care, internal medicine, hepatology, gastroenterology, endocrinology, obesity medicine and drug development.

Despite substantial progress, MASLD is still a leading cause of chronic liver disease, including primary liver cancer [6]. The “multiple hit” hypothesis has emerged as the dominant theory in recent years, indicating the collective impact of immunological mechanisms in the liver, intestines, and adipose tissue on the progression of MASLD [13]. Overnutrition with a poor-quality diet rich in glucose, high-fructose corn syrup and saturated fat leads to increased intrahepatic triglyceride levels, which are characteristic of MASLD, through multiple pathways [14]. Accumulating evidence supports the concept that ballooned hepatocytes, a defining feature of steatohepatitis, generate a secretome that induces inflammatory and fibrogenic responses in neighboring cells [15]. Both innate and adaptive immune cells play indispensable roles in the development and progression of inflammation in MASLD [16]. Interestingly, there are sex and sex differences in the prevalence of MASLD, and potential sex differences in innate immune cell types have been suggested to affect its development [17]. Most importantly, immune cell-mediated inflammation plays a key role in simple steatosis-to-MASH conversion. Recently, multiple immune-related mechanisms have been revealed, leading to the identification of several promising targets. For example, the expression of the immune regulator PU.1 is increased in the liver, primarily in macrophages, of obese mice and people, and pharmacologic PU.1 inhibition results in improved metabolic dysfunction in HFD-fed mice [18]. In addition to PU.1, the expression of EF-hand domain family member D2 (EFHD2) was also significantly increased in hepatic macrophages/monocytes from both patients and mice with MASH, which contributed to liver macrophage/monocyte reprogramming in MASH, whereas targeted EFHD2 inhibition via a hydrocarbon-stapled peptide effectively attenuated disease in mice [19]. Moreover, the targeting of Th17 cells is promising for the treatment of MASLD. Ursolic acid was shown to target secreted phosphoprotein 1 (SPP1) to regulate Th17 cells, thus improving immune inflammation in MASLD [20].

**Fig. 1:**
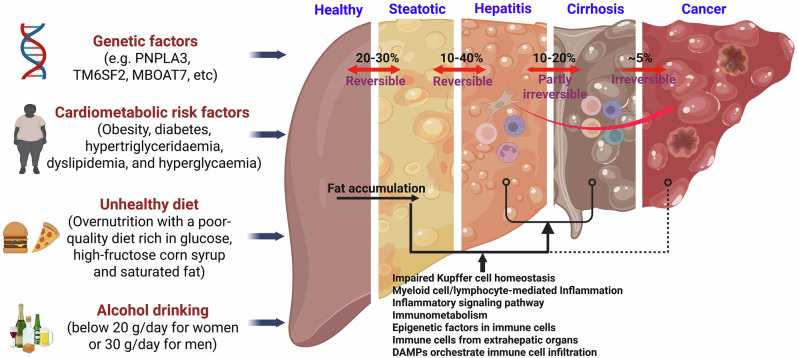
A spectrum of MASLD. MASLD represents a spectrum of disorders ranging from simple steatosis to MASH, ultimately resulting in advanced fibrosis, cirrhosis and cancer. Immunological mechanisms are involved in the development of MASLD, especially simple steatosis-to-MASH conversion

Notably, MASH could soon be the top indication for liver transplantation across all subgroups if the current estimates for the increase in MASH remain [21]. HCC is the terminal stage in the progression of MASLD. Emerging studies have shown that immune regulation is involved in the transition from MASH to HCC. For example, fibroblast growth factor 21 (FGF21) exhibits important anti-inflammatory activity, and its deficiency can induce excessive KC death and accelerate the MASH-HCC transition [22]. Additionally, interleukin-21 (IL-21) is a cytokine that is produced primarily by T cells and natural killer T cells, and the IL-21 receptor (IL-21R) is a specific receptor. IL-21R signaling has been shown to promote MASH-HCC via the induction of immunosuppressive IgA^+^ B cells [23]. Therefore, understanding the contribution of immunological mechanisms in MASLD may be important for identifying novel therapeutic approaches for this disease, regardless of whether it is in the early or late stage. In this review, we describe the emerging data regarding the immunological mechanisms of MASLD (Fig. 1) and summarize data regarding new concepts related to immunoregulation-based therapies for the treatment of MASLD.

## Immunological mechanisms of MASLD

### Macrophage populations and phenotypes in MASLD

Immunological mechanisms in MASLD involve the concerted actions of tissue-resident and recruited immune cells. Liver macrophages play a central role in MASLD, influencing inflammation, fibrosis progression, and resolution [24, 25]. In human liver biopsies, the accumulation of macrophages is closely associated with the severity of MASH and fibrosis [26]. Over the past decade, high-resolution methods, such as single-cell RNA sequencing (scRNAseq), single-nuclear RNA sequencing (snRNAseq), spatial transcriptomics, and spatial proteogenomics, have substantially increased our knowledge of different macrophage subsets [27]. While the dominant (>90%) macrophage population in a healthy liver is embryonic-derived Kupffer cells (EmKCs), also known as resident Kupffer cells (ResKCs) (more specifically, below), these cells are progressively eliminated during MASLD and are replaced by bone marrow-derived circulating monocytes, also known as monocyte-derived macrophages (MdMs) [28].

**Fig. 2:**
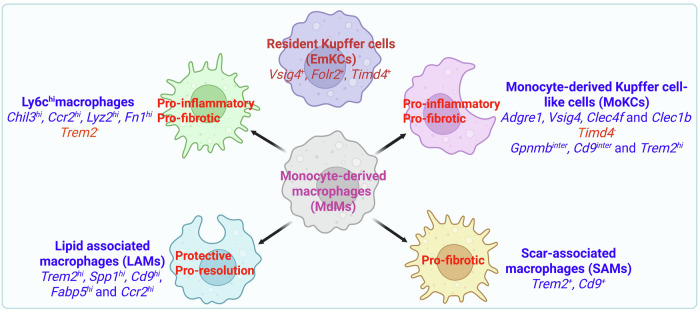
Major subtypes of liver macrophages based on their transcriptomic signature in MASLD. Liver macrophages include resident Kupffer cells (EmKCs) and monocyte-derived macrophages (MdMs). MdMs can infiltrate the liver and can be further classified into different MASH-associated macrophage subsets, which play critical roles in MASLD development and progression

Dynamic changes in macrophage heterogeneity are a defining feature of MASLD that includes their distinct ontogeny and functional phenotype [28]. MdMs in the MASH liver can be further classified into the following major subtypes on the basis of their transcriptomic signature [25]: (i) *Ly6C*^*hi*^-recruited macrophages (which are also *Chil3*^*hi*^*, Ccr2*^*hi*^*, Lyz2*^*hi*^*, and Fn1*^*hi*^) and *Trem2* negative. These macrophages are proinflammatory and profibrotic. (ii) Monocyte-derived cells occupying the Kupffer cell niche (MoKC). This subpopulation of cells expresses several EmKC markers (such as *Adgre1, Vsig4, Clec4f* and *Clec1b*) but does not express *Timd4*. These cells also express intermediate levels of *Gpnmb* and *Cd9*. Notably, this subpopulation also highly expresses *Trem2*. (iii) Lipid-associated macrophages (LAMs), defined as *Trem2*^*hi*^*, Spp1*^*hi*^*, Cd9*^*hi*^, *Fabp5*^*hi*^ and *Ccr2*^*hi*^. Both MoKC and LAMs express disease protection and proresolution gene signatures (Fig. 2). Interestingly, a small population of LAMs, known as bile duct LAMs (BD-LAMs), are also found in healthy livers and are localized surrounding the bile ducts [29]. In addition to MASH, Trem2^+^ LAMs have been identified in various liver injury models, including acetaminophen (APAP)- and carbon tetrachloride (CCl_4_)-induced liver damage, where they play crucial roles in the efficient clearance of dying cells [30].

### Lipid-associated macrophages and their roles in MASLD

In MASH livers, Trem2^+^Gpnmb^+^ hepatic LAMs are often found in hepatic crown-like structures (hCLS) [28]. However, the precise roles of hepatic LAM and hCLS in MASH development remain unclear. Determining whether immune cells associated with hCLS exert protective or pathogenic effects is important. *Ccr2*^*−/−*^ mice, which lack hepatic LAMs, exhibit exacerbated MASH [28]. The absence of Trem2 also reduces the emergence of LAM and hCLS during both disease progression and regression, which is correlated with worse disease outcomes [25]. Trem2^+^CD9^+^ macrophages were also found to be associated with fibrotic scars and were named scar-associated macrophages (SAMs), suggesting a profibrogenic role [31].

TREM2 was first cloned from human PBMCs in 2000 [32, 33]. Its function in macrophages was first identified in 2006, revealing that Trem2 plays a crucial role in restraining macrophage activation [34]. In 2019, via index and transcriptional single-cell sorting, a novel and conserved Trem2^+^ LAM subset was identified in both mouse and human livers during obesity [35]. This specific subset has a transcriptional signature of *Trem2*, *Lipa*, *Lpl*, *Ctsb*, *Ctsl*, *Fabp4*, *Fabp5*, *Lgals1*, *Lgals3*, *Cd9*, and *Cd36*, which are closely associated with lipid metabolism and phagocytosis. Importantly, genetic ablation of *Trem2* in obese mice suppressed the downstream molecular LAM program, resulting in adipocyte hypertrophy, systemic hypercholesterolemia, body fat accumulation and glucose intolerance [35], providing a new perspective on the function of Trem2 in metabolic disease. A Trem2^+^ MASH-associated macrophage subset, which is a feature of mouse and human MASH that is linked to disease severity, was subsequently identified [36]. Spatial transcriptomics further revealed that Trem2^+^ macrophages localize to sites of hepatocellular damage, inflammation and fibrosis in the steatotic liver [37]. Strikingly, *Trem2*-deficient macrophages release exosomes that impair hepatocytic mitochondrial structure and energy supply due to their high content of miR-106b-5p, which blocks Mitofusin 2 (Mfn2) expression in MASLD [38]. Conversely, the overexpression of Trem2 in macrophages improved MASLD, suggesting that Trem2 is a potential target for MASLD treatment by regulating hepatocyte‒macrophage metabolic coordination [38]. Trem2 is required for the macrophage-dependent efferocytosis of lipid-laden apoptotic hepatocytes. In the MASH liver, prolonged hypernutrition triggers a surge of proinflammatory cytokines, such as TNFα and IL-1β, which in turn activate ADAM17, an enzyme responsible for cleaving Trem2 from the surface of macrophages. This shedding of Trem2 compromises the ability of macrophages to perform efferocytosis, the critical process of clearing dead cells and debris. Consequently, the impaired removal of cellular waste perpetuates chronic inflammation, fostering a vicious cycle that fuels MASH pathology [39].

How infiltrating macrophages acquire the LAM gene signature, including Trem2 expression, is currently being investigated. A MASH-inducing diet induced changes in chromatin accessibility, H3K27ac modifications, and genome-wide LXR (liver X receptor) and ATF3 (activating transcription factor 3) binding, leading to the reprogramming of LXR activity in liver macrophages. ATF3 recruits LXRs to AP-1 motif-containing enhancers, enabling the activation of genes such as *Trem2* and *Cd9*, suggesting their role in shaping SAM and LAM phenotypes [40]. Hepatocyte-derived sphingosine-1 phosphate (S1P) was also shown to induce Trem2 expression in macrophages [39].

While macrophage heterogeneity in MASH progression is well characterized, the fate of MASH-associated macrophage populations during regression remains unclear. A recent study indicated that macrophage heterogeneity in livers undergoing MASH regression closely mirrors that of active MASH [25]. Although no new macrophage subpopulations emerge during regression, the distribution and dynamics of existing macrophage subpopulations undergo significant changes compared with those of active MASH. Interestingly, the EmKCs that were lost during MASH did not return to normal levels within the time frame of regression [25], and whether they would return to normal homeostatic levels needs further investigation. The regression livers were dominated by the LAM subpopulation [25, 41]. Importantly, Trem2^+^ LAMs constitute the key macrophage subpopulation that drives MASH-related fibrosis resolution and tissue repair. A lack of Trem2 macrophages prevents elimination of hepatic steatosis and resolution of fibrosis during dietary intervention-mediated regression [25]. This phenomenon was also observed in bariatric surgery-mediated MASH regression [41], reinforcing the importance of TREM2^+^ LAMs in MASH resolution.

Given the strong induction of Trem2 expression during MASH, circulating levels of soluble Trem2 (sTrem2) in MASH were demonstrated to correlate with disease severity in mice and humans [37]. Plasma sTrem2 levels are associated with individual histologic features of NAFLD activity scores (NASs), such as steatosis, lobular inflammation, and ballooning, but only weakly with fibrosis stage, suggesting that sTrem2 in plasma may be a noninvasive biomarker that can rule-in or rule-out the presence of MASH with elevated liver stiffness [42]. Furthermore, in a multicenter proteomic study, a composite model comprising four proteins (ADAMTSL2, AKR1B10, CFHR4 and Trem2), body mass index and type 2 diabetes mellitus status was used to identify at-risk MASH [43]. These findings suggest that sTrem2 could be a useful biomarker in the management of MASH.

In addition to these investigations on Trem2^+^ LAMs during MASLD, scRNA-seq analysis identified other immune cell subsets, such as brain-abundant membrane-attached signal protein 1 (Basp1)^+^ myeloid cells and membrane-spanning 4-domains a7 (MS4A7)^+^ macrophages. Basp1 is a myeloid-enriched gene that was significantly elevated in the livers of mouse and human MASH. Myeloid-specific inactivation of Basp1 ameliorated MASH by diminishing the response to proinflammatory stimuli, impairing NLRP3 inflammasome activation and blocking cytokine secretion [44]. Steatotic hepatocytes release lipid droplets (LDs), which promote MASH-related liver injury in an MS4A7-dependent manner. MS4A7, a Trem2^+^ MASH-associated macrophage-specific pathogenic factor, exacerbated MASH progression in mice by inducing NLRP3 inflammasome activation via direct physical interaction, indicating that the LD-MS4A7-NLRP3 inflammasome axis is a key pathogenic driver of MASH progression [45].

### Role of resident Kupffer cells and infiltrating macrophages

The healthy liver harbors a large population of resident macrophages called embryonic Kupffer cells (EmKCs), which are derived from the yolk sac during embryogenesis. EmKCs are located primarily in the hepatic sinusoids and act as professional phagocytes to phagocytose microbes, including bacteria, viruses and fungi, alongside damaged cells. Owing to recent technological advances, including the application of scRNAseq and spatial transcriptomics to mouse models, we now know that the population of EmKCs is depleted during MASLD development and is replaced by distinct subsets of bone marrow-derived macrophages [29, 46]. The reduction in EmKCs in the liver is partly due to increased Kupffer cell (KC) death during MASLD development. However, EmKC death is not required for bone marrow-derived macrophage infiltration in MASLD [47]. The molecular mechanisms underlying KC loss are poorly understood. Hypoxia-inducible factor 2α (HIF-2α) plays a crucial role in controlling KC death in MASLD, as evidenced by the fact that HIF-2α decreases lysosomal and phagocytic gene expression in KCs by inducing mammalian target of rapamycin (mTOR)- and extracellular signal-regulated kinase-dependent inhibitory transcription factor EB (TFEB) phosphorylation [48]. These changes were sufficient to increase lysosomal stress, resulting in decreased efferocytosis and lysosomal cell death. In addition, monocyte-derived macrophages, resembling monocyte-derived KCs (MoKCs), gradually seeded into the KC pool. Furthermore, these MoKCs are partly immature and exhibit a proinflammatory status compared with EmKCs [49]. Interestingly, HIF-2α-dependent TFEB regulation occurred only in KCs but not in MoKCs [48].

Fibroblast growth factor 21 (FGF21), an endocrine FGF primarily produced in the liver, protects against MASH [50]. Interestingly, FGF21 plays a critical role in maintaining the KC pool by regulating sphingosine-1-phosphate (S1P)-YAP signaling, thus preventing the progression of MASH to hepatocellular carcinoma [22]. Notably, CLEC4F in mice is a common marker for KCs but is not a good marker for EmKCs in MASLD since MoKCs also express CLEC4F. Therefore, a range of conserved markers, including VSIG4, TIM4, and FOLR2, may help to distinguish EmKCs from MoKCs in MASLD [51]. Recently, two subsets of EmKCs were identified in MASLD, including a major CD206^lo^ESAM^-^ population (KC1) and a minor CD206^hi^ESAM^+^ population (KC2), which have specific transcriptomic and proteomic signatures. Functionally, only KC2 is involved in the regulation of liver metabolism in MASLD via elevated expression of the fatty acid transporter CD36 [52]. However, the identity of KC2 is currently debated in the field because of its close transcriptomic profile to that of liver sinusoidal endothelial cells (LSECs) [51, 53].

KCs are iron storage cells that play a key role in iron metabolism [54]. Iron overload can induce ferroptosis, which is characterized by lipid peroxidation [55]. A recent study determined the significance of neutrophil cytosolic factor 1 (NCF1)-mediated KC ferroptosis in human and mouse MASH [56]. Macrophage NCF1 induced iron overload and ferroptosis in KCs by increasing the level of oxidized phospholipids, which promoted TLR4-dependent hepatocyte hepcidin production, thereby exacerbating MASH progression. These findings suggest that impaired KC homeostasis is essential for MASLD development and progression.

**Fig. 3:**
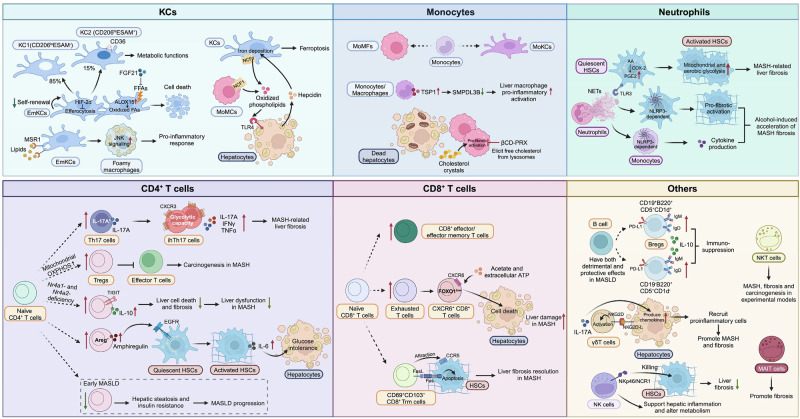
The diverse roles of different immune cells in MASLD. Two subsets of EmKCs are identified in MASLD, including a major CD206^lo^ESAM^-^ population (KC1) and a minor CD206^hi^ESAM^+^ population (KC2), which have specific transcriptomic and proteomic signatures. Neutrophil-derived NETs exacerbate MASH-related liver fibrosis by inducing metabolic reprogramming of HSCs. NETs accelerate alcohol-induced acceleration of MASH-related liver fibrosis by activating HSCs and monocytes via NLRP3. Lymphocytes, including T cells, B cells, and innate lymphoid cells, play critical and diverse roles in MASLD

The majority of hepatic MASH macrophages are derived from recruited bone marrow-derived macrophages, which have diverse functions, as they adapt their phenotype (“polarization”) in response to the local environment [57]. To visualize these cells and processes, positron emission tomography (PET) imaging of recruited macrophages by targeting CCR2 and CD163 has translational potential for the diagnosis and monitoring of MASH [58]. Macrophages affect MASH through the release of different factors. For example, macrophage-derived thrombospondin 1 (TSP1) significantly contributes to MASH development and progression, as macrophage-specific TSP1 deletion protects mice against obesity-associated liver injury, inflammation and fibrosis [59]. Mechanistically, TSP1 amplified Toll-like receptor 4 (TLR4) proinflammatory signaling by inhibiting *Smpdl3b* expression in the liver. Since recruited macrophages can take up fatty acids, the underlying mechanism by which fatty acids affect macrophage function in MASLD remains incompletely understood. Macrophage scavenger receptor 1 (MSR1), an innate immune receptor, plays a key role in the clearance of circulating lipoproteins and has been investigated in inflammatory disorders [60]. A recent study demonstrated that MSR1 mediated lipid uptake and accumulation in macrophages, leading to liver inflammation and thus accelerating MASLD. MSR1 induced a proinflammatory response via the JNK signaling pathway in macrophages. Macrophage-specific *Msr1*-deficient mice are resistant to diet-induced metabolic disorders, as evidenced by fewer hepatic foamy macrophages, reduced inflammation, improved glucose tolerance and hepatic lipid metabolism [61], suggesting that blocking MSR1 signaling could be a promising approach for the treatment of MASLD. Lipotoxic lipids, such as free cholesterol, not only induce hepatocyte death but also trigger lysosomal dysfunction and profibrotic activation of macrophages during MASH development [62]. β-Cyclodextrin polyrotaxane (βCD-PRX), a unique supramolecule, is able to elicit free cholesterol from lysosomes. Treatment with βCD-PRX effectively mitigated MASH-related liver fibrosis by decreasing cholesterol accumulation and the profibrotic activation of macrophages surrounding dead hepatocytes with cholesterol crystals without affecting hepatic and serum levels of cholesterol [62]. Taken together, the hepatic macrophage landscape in MASLD is highly heterogeneous, consisting of a combination of resident and recruited macrophages of different subsets. Understanding the functional roles of these subsets may help in developing concepts to efficiently target macrophages for MASLD treatment (Fig. 3).

### Role of neutrophils

Neutrophils are the most abundant leukocytes in the blood and form a critical arm of innate immunity, playing a well-recognized role in antibacterial defense by extruding a meshwork of chromatin fibers to establish neutrophil extracellular traps (NETs) via a process known as NETosis [63, 64]. However, neutrophils can also be pathogenic by releasing various inflammatory mediators, including different cytokines, myeloperoxidase (MPO), reactive oxygen species (ROS), and proteases [65]. The role of neutrophils has garnered attention for their involvement in sterile inflammation, which plays a pivotal role in the pathogenesis of steatotic liver disease [66]. Lipotoxic hepatocytes release chemokines (CXCL1 and IL-8) that recruit neutrophils to the liver [64]. Hepatic overexpression of *Cxcl1* and *IL8* accelerated the transition from steatosis to MASH in HFD-fed mice and was associated with marked neutrophil infiltration and hepatocyte death [67]. Additionally, neutrophil-derived NETs exacerbated MASH-related liver fibrosis by inducing metabolic reprogramming of HSCs. NETs induce HSC activation, proliferation, and migration through increased mitochondrial and aerobic glycolysis to provide additional bioenergetic and biosynthetic supplies via the toll-like receptor 3/cyclooxygenase-2 pathway [68]. In addition, NETs accelerated alcohol-induced acceleration of MASH-related liver fibrosis by activating HSCs and monocytes via Nod-like receptor protein 3 (NLRP3) [69]. These studies highlight the potential of NET inhibition as a novel therapeutic strategy to combat MASH-related liver fibrosis (Fig. 3).

Neutrophil elastase (NE), a serine protease secreted by neutrophils during inflammation via the degranulation process, plays a significant role in the pathogenesis and progression of MASH [70]. The NE that is released into the extracellular space is bound by alpha-1-antitrypsin (AAT), which serves as an inhibitor. Consequently, the serum NE or NE-to-AAT ratio is commonly used as a biomarker to assess MASLD severity [71]. Knockout of *Elane*, which encodes NE, protects against Western diet-induced MASH [70]. In summary, neutrophil elastase plays a critical role in NASH pathogenesis by driving liver injury, inflammation, and fibrosis. Its potential as both a biomarker and therapeutic target highlights the need for further investigation in MASH management.

### Role of lymphocytes

An increasing number of studies have revealed that lymphocytes play critical and diverse roles in MASLD. Lymphocytes include T cells, B cells, and innate lymphoid cells. There are different populations of CD4+ T cells, such as Th1, Th2, Th17, and Foxp3^+^ regulatory T (Treg) cells. Although a role of CD4^+^ T cells in MASLD has been suggested [72], the exact polarization state of these cells during MASLD development remains obscure. Single-cell RNA sequencing and multiple-parameter flow cytometry were used to identify a population of multicytokine-producing CD4^+^ T cells. Interestingly, among these cells, only those with a Th17 polarization state were enriched in patients with MASH-related fibrosis, providing the rationale to target CD4^+^ T cells with a Th17 polarization state to inhibit MASH-related fibrosis development [73]. Similarly, a distinct population of inflammatory hepatic CXCR3^+^ Th17 cells emerges in MASLD, which are sufficient to increase chromatin accessibility, glycolytic skewing and concomitant production of IL-17A, IFNγ, and TNFα, thus exacerbating MASLD pathogenesis [74]. Although Tregs support tissue function and tissue homeostasis by limiting inflammation and promoting repair [75], the functions of Tregs in MASLD initiation and progression are not fully understood. This may be due to the varying effects of Tregs in response to different stimuli and the corresponding microenvironments in different stages of MASLD. For example, an interesting study revealed that the Treg subpopulation was selectively elevated in the liver of MASH patients, despite a decreased number of CD4^+^ T cells. This increase in Tregs accelerated MASH, as evidenced by the fact that depleting Tregs dramatically inhibited MASH progression [76]. Another study demonstrated that a massive accumulation of Tregs in the livers of MASH mice ameliorated liver cell death and fibrosis, thus mitigating liver dysfunction in MASH [77]. Nevertheless, the number of Tregs was found to be decreased in early MASLD, contributing to high-fat diet-induced hepatic steatosis [78]. Indeed, it is inherently difficult to dissect the exact role of Tregs in MASLD by depleting Tregs in mice. Examining the effects of Treg-derived mediators may reveal the roles of Tregs in liver diseases. For example, amphiregulin (Areg)-producing Tregs are enriched in the livers of mice and humans with MASH. *Areg* deficiency in Tregs blocked MASH-related liver fibrosis. Mechanistically, Areg can activate profibrotic transcriptional programs in hepatic stellate cells via epidermal growth factor receptor (EGFR) signaling and promote hepatocyte gluconeogenesis via IL-6 [79]. Overall, more studies are needed on the different populations of Tregs in different stages of MASLD to identify specific Treg-derived pathogenic contributions.

Many studies have investigated the functions of CD8^+^ T cells, B cells, and innate lymphoid cells in MASLD. In detail, activated clonally expanded CD8^+^ T cells, which have a transcriptional profile related to chronic antigenic stimulation, significantly accumulate in the livers of MASH mice and patients with MASH-related cirrhosis, indicating a potential role for antigen activation of CD8^+^ T cells in MASH pathogenesis [80]. In addition, liver-resident CXCR6^+^CD8^+^ T cells were found to be abundant in MASH mice and patients with MASH. These specific CD8^+^ T cells are more susceptible to metabolic stimuli (including acetate and extracellular ATP), triggering autoaggression in an IL-15-induced FOXO1-dependent manner during MASH development [81]. Accordingly, single-cell transcriptome analysis revealed that CD69^+^CD103^-^CD8^+^ tissue-resident memory (Trm) cells were enriched in the livers subjected to MASH resolution, which attracted hepatic stellate cells in a CCR5-dependent manner and resulted in HSC apoptosis, thereby promoting MASH-related liver fibrosis resolution [82].

B cells have both detrimental and protective effects on MASLD, and their roles in MASLD remain controversial [83]. B-cell deficiency mitigated MASH progression, and adoptively transferring B cells for MASH livers recapitulated the disease [84]. Furthermore, another study demonstrated that B-cell-depleted mice were only partially protected from MASH, whereas B-cell-harboring but antibody-deficient *IgMi* mice were fully resistant to MASH [83]. Strikingly, increases in two regulatory B-cell subsets, CD19^+^B220^+^CD5^+^CD1d^+^ and CD19^-^B220^+^CD5^+^CD1d^-^, were observed in the livers of mice subjected to MASLD. These specific B-cell subsets express PD-L1, IL-10, IgM, and IgD, thus exerting immunosuppressive effects, especially CD19^-^B220^+^CD5^+^CD1d^-^ B cells [85]. Lipid accumulation in hepatocytes induces the expression of ligands specific to the activating immune receptor NKG2D, which activates tissue-resident innate-like T cells, most notably γδ T cells, leading to IL-17A secretion. IL-17A subsequently promotes chemokine production in hepatocytes, which recruit proinflammatory cells into the liver, ultimately accelerating MASH [86].

Innate lymphoid cells and unconventional T cells represent emerging but incompletely understood contributors to MASLD pathobiology. Innate lymphoid cells (ILCs), which are based on developmental and functional trajectories, can be divided into five subsets: natural killer (NK) cells, ILC1s, ILC2s, ILC3s, and lymphoid tissue inducer (LTi) cells [87]. Data on ILCs are emerging and partially controversial. For example, NK cells have long been considered antifibrogenic because of their potential to eliminate hepatic stellate cells. Mechanistically, NKp46/NCR1, a specific marker of NK cells, mediates the killing of human and mouse HSCs through unknown ligands, thus resulting in the attenuation of liver fibrosis [88]. However, in the context of obesity, NK cells may also support inflammation and alter metabolism via the induction of hepatic ER stress via osteopontin production and other mechanisms [89]. Thus, NK cells may have both protective and deleterious effects on MASLD, depending on the stage of the disease and the microenvironment.

Unconventional T cells are a heterogeneous group of lymphocytes that are abundant in the liver (they represent the majority of intrahepatic T cells but only 10% of T cells in the blood). The most important subsets of unconventional T cells include natural killer T (NKT) cells, γδ T cells, and mucosal-associated invariant T (MAIT) cells [90]. NKT cells are much more abundant in mouse livers than in human livers, and they promote MASH, fibrosis, and carcinogenesis in experimental models [91]. Given the opposing distributions of NKT cells and MAIT cells between human and mouse livers, findings about NKT cells from rodent models need to be interpreted with caution, and MAIT cells have been suggested as the human counterpart of NKT cells from a functional perspective [92]. The role of MAIT cells in the liver is evolving. Experimental evidence supports profibrogenic functions in chronically injured livers, and inhibiting MAIT cells promotes fibrosis regression via the induction of a repair phenotype in hepatic macrophages [93–95]. Taken together, these findings highlight the diverse roles of lymphocytes in MASLD (Fig. 3).

### Immunometabolism in macrophages

More than a decade ago, the concept of immunometabolism was proposed to elucidate the intricate interplay between metabolism and immunity [96]. Metabolism, including lipid metabolism, fatty acid oxidation, oxidative phosphorylation, glycolysis, and other metabolic pathways, influences the differentiation and function of immune cells. Accelerating evidence suggests that the metabolic reprogramming of immune cells plays essential roles in the pathogenesis of MASLD [97].

Among the two distinct populations of Kupffer cells mentioned above, i.e., a major CD206^lo^ ESAM^-^ population (KC1) and a minor CD206^high^ ESAM^+^ population (KC2), only KC2-expressing genes are involved in liver metabolism regulation, including fatty acid metabolism both in steady-state and in diet-induced obesity in mice, as well as steatosis induction via the fatty acid transporter CD36 [52]. In addition to lipid metabolism in Kupffer cells, the activation of G-protein coupled receptor 3 (GPR3) in Kupffer cells promotes glycolysis via the formation of complexes between β-arrestin 2 and key glycolytic enzymes, ultimately inhibiting inflammation and ameliorating MASLD [98]. These findings suggest that metabolic reprogramming of Kupffer cells is a pivotal mechanism in MASLD.

**Fig. 4:**
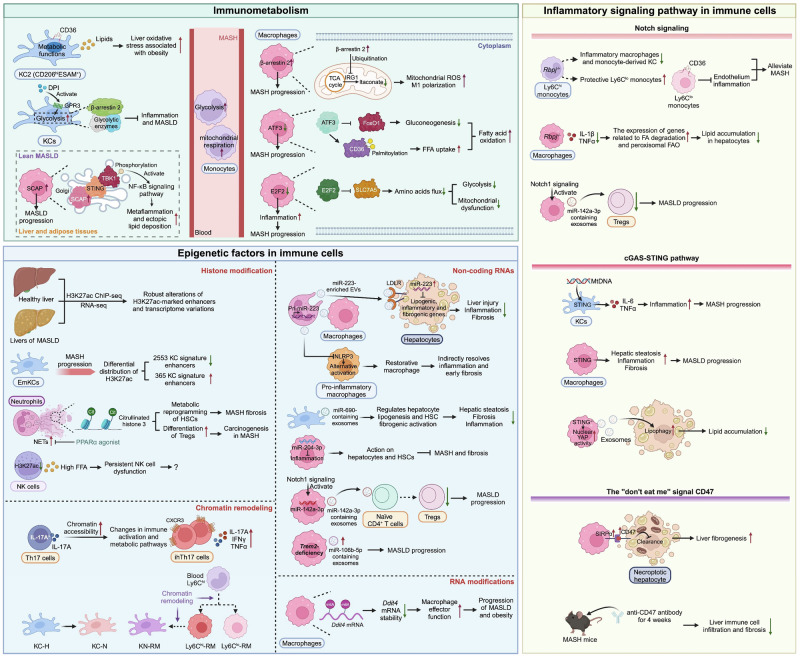
Other immunological mechanisms of MASLD. Metabolic reprogramming, epigenetic regulation and inflammatory signaling pathways in immune cells play essential roles in the pathogenesis of MASLD

Moreover, the metabolic reprogramming of monocyte-derived macrophages has attracted much interest because of its importance and heterogeneity. β-Arrestin 2, a multifunctional adaptor protein for the desensitization and internalization of G protein-coupled receptors (GPCRs) [99], was found to be elevated in liver macrophages and circulating monocytes in patients with MASH. Interestingly, β-arrestin 2 in myeloid cells promoted the ubiquitination of immune responsive gene 1 (IRG1), leading to decreased itaconate production and increased succinate dehydrogenase activity in macrophages. These dysregulated Krebs cycle metabolites fuel the release of mitochondrial reactive oxygen species and inflammatory polarization, ultimately exacerbating MASH. Targeting β-arrestin 2 in myeloid cells may be a potential strategy for MASH treatment [100]. Activating transcription factor 3 (ATF3), a stress-induced transcription factor that binds to the cyclic AMP response element (CRE), plays a vital role in metabolic regulation and immunity [101]. Intriguingly, ATF3 protein expression in liver macrophages was downregulated and negatively correlated with MASH score. Importantly, ATF3 is a regulator of the glucose‒fatty acid cycle and promotes fatty acid oxidation in macrophages via the reduction of cellular glucose levels via the inhibition of head box 1 (FoxO1)-mediated gluconeogenesis and the induction of free fatty acid (FFA) uptake by CD36, suggesting that ATF3 improves glucolipid metabolism in liver macrophages, ultimately protecting against MASH development [102]. These studies suggest that correcting aberrant metabolic pathways in macrophages may be a plausible approach for MASLD (Fig. 4).

Given the importance of energy metabolism, an increasing number of studies have investigated the bioenergetic profile of immune cells in MASLD. A recent study revealed a distinct bioenergetic phenotype in the circulating monocytes of mouse MASH models and patients with MASH, characterized by the upregulation of the mitochondrial and glycolytic energy pathways [103]. The increase in mitochondrial respiratory chain activity in macrophages results in an imbalance in the production of ROS and cytokines, thus accelerating MASLD. E2F2, a crucial member of the E2F family, plays an important role in regulating cell proliferation, differentiation and apoptosis [104]. Recently, the expression of E2F2 in macrophages was found to be dramatically downregulated in MASH. This downregulation exacerbated MASLD progression, as demonstrated by the finding that *E2f2* deficiency in macrophages aggravated liver inflammation, hepatic stellate cell activation and lipid accumulation. A mechanistic study further revealed that *E2f2* deletion in macrophages fueled proinflammatory responses in a Leu-mTORC1-dependent manner by enhancing glycolysis and impairing mitochondrial function by increasing SLC7A5 expression [105].

Immunometabolism is involved not only in obese MASLD but also in lean MASLD. Sterol regulatory element binding protein cleavage-activating protein (SCAP) is a cholesterol sensor that functions to affect intracellular cholesterol homeostasis [106]. During lean MASLD development, *Scap* expression in macrophages is abnormally elevated in adipose and liver tissues. Moreover, macrophage-specific *Scap* deletion ameliorated metainflammation and ectopic lipid deposition by promoting the release of inflammatory mediators by recruiting STING and tank-binding kinase 1 to the Golgi to activate the NF-κB signaling pathway [107]. Nevertheless, whether SCAP influences obese MASLD development and progression remains unclear.

### Epigenetic factors in immune cells

Epigenetic regulation is a mechanism that modulates heritable gene expression without altering the DNA sequence and includes chromatin remodeling, histone modifications, and noncoding RNA (ncRNA) regulation, among other mechanisms [108]. Histone modification is one of the most thoroughly examined mechanisms involved in controlling the function of immune cells in MASLD (Fig. 4). Genome-wide profiling of the acetylation of histone H3 lysine 27 (H3K27ac), a marker of active enhancers and promoters, revealed that H3K27ac density is significantly altered in the livers of MASLD rats, along with abundant alterations in the regulatory transcriptome [109]. Interestingly, H3K27ac is involved in the epigenetic change in resident KCs during MASH, as the MASH diet significantly downregulates 2553 enhancers related to the KC signature, whereas only 365 KC signature enhancers are upregulated, which is demonstrated by the differential distribution of H3K27ac, suggesting a vital role of histone acetylation in reprogramming the KC enhancer landscape under MASH conditions [40]. In addition to macrophages, numerous studies have shown that histone modifications in neutrophils are also essential for the development and progression of MASLD through the formation of neutrophil extracellular traps (NETs). NETs are weblike structures that contain DNA, histones and various granule proteins, serving as robust defenses against microbes. In NETs, histone 3 citrullination is considered an early event in NET formation, and it is well acknowledged as a NET-specific marker [110]. Neutrophil infiltration and NET formation are increased during the development of MASLD [68, 69], and NETs reportedly promote MASH by inducing metabolic reprogramming of HSCs [68]. In addition, NETs are able to promote the differentiation of regulatory T cells (Tregs), which contribute to carcinogenesis in MASLD [76], whereas activating PPARα can reduce NET formation in a high-fat diet (HFD) context [111]. Additionally, lipid accumulation results in persistent NK cell dysfunction via histone hypoacetylation, and impaired functions are also observed in liver NK cells from HFD-fed mice [112].

Moreover, the functions of noncoding RNAs (ncRNAs), particularly microRNAs (miRNAs), have been widely studied in MASLD [113, 114]. Extracellular vesicles (EVs) are membrane-derived vesicles that facilitate cellular crosstalk. Neutrophils can produce various subtypes of EVs containing proinflammatory miRNAs or anti-inflammatory miRNAs [115]. Among these miRNAs, miR-223 is broadly recognized as a protective factor in MASLD via myeloid cell-to-hepatocyte communication by targeting several inflammatory genes and oncogenes in hepatocytes to prevent MASH and MASLD-HCC [116, 117]. In addition, it has been reported that miR-223 also mediates the function of neutrophils in spontaneous resolution of liver fibrosis [118]. In addition to miR-223, miR-690 was also shown to alleviate MASLD. KCs are considered the dominant source of miR-690 in the liver, and miR-690 is important for normal KC function. Treating MASH mice with miR-690 inhibited hepatic steatosis, fibrosis and inflammation [119]. In addition, miR-204-3p in macrophages was also demonstrated to be a protective factor in MASLD progression by ameliorating macrophage inflammation and coordinating macrophage actions on hepatocytes and HSCs to alleviate steatohepatitis and fibrosis [120]. miR-142a-3p is another miRNA that is essential for the functions of macrophages, whose secretion is regulated by Notch1 signaling. However, miR-142a-3p may contribute to MASLD by impeding Treg differentiation [78]. Importantly, the livers of patients with MASLD and mouse MASLD models display a distinct population of TREM2^+^ macrophages, and *Trem2* deficiency in macrophages exacerbates mitochondrial dysfunction in hepatocytes by releasing exosomes containing high levels of miR-106b-5p, leading to MASLD progression [38]. Overall, macrophages can release miRNA-containing EVs to exert their effects on other cells, including hepatocytes and Tregs; conversely, the activation of macrophages is also regulated by other liver cells, such as hepatocytes. For example, miR-28-5p secreted by hepatocytes stimulates the expression of inflammatory factors in macrophages to aggravate MASH [121]. Collectively, these results reveal that miRNAs are pivotal mediators of the functions of immune cells in MASLD (Fig. 4).

Notably, with the help of an assay for transposase-accessible chromatin using sequencing (ATAC-seq), chromatin remodeling in immune cells during the development and progression of MASLD, especially chromatin accessibility, has been increasingly revealed. Compared with those in the conventional hepatic Th17 cell cluster, inflammatory hepatic Th17 (ihTH17) cells accumulate in mice with diet-induced obesity, whose chromatin accessibility is increased in the regulatory regions of genes associated with immune activation and metabolic pathways. Furthermore, hepatic accumulation of ihTh17-like cells is positively correlated with MASLD severity in humans, suggesting that the metabolic skewing of hepatic Th17 cells by epigenetic regulation is critical in the pathogenesis of MASLD [74]. Additionally, changes in chromatin accessibility also contribute to the diversity of myeloid cells in MASH. Myeloid diversity has been investigated in mice fed a control diet or MASH diet, and the three most abundant clusters (KC-H, healthy KC; KC-N, NASH KC; and KN-RM) are recruited macrophages occupying the KC niche. In addition to the main macrophage cluster, two other clusters exist. Specifically, Ly6C^hi^-RM refers to macrophages that display relatively high levels of transcripts typical of Ly6C^hi^ monocytes, whereas Ly6C^lo^-RM expresses high amounts of *Cd209a*, *Cd7* and *Itgax*. In contrast to the obvious change in H3K27ac deposition during the transition from KC-H to KC-N, the transition of Ly6C^hi^ blood monocytes to KN-RM or Ly6C^lo^-RM is thought to be directed by chromatin remodeling to modulate the open chromatin regions [40]. Therefore, changes in chromatin accessibility are essential for the reprogramming and activities of immune cells. However, whether other mechanisms, including chromatin structure and chromatin stability, are involved in the role of immune cells in MASLD is largely unknown, but newer technologies, such as 5 C, Hi-C, Micro-C, DNAse-Hi-C, Chia-PET, HiChIP, and Plac-seq, are expected to decipher the understanding of chromosome conformation [122].

Other epigenetic mechanisms, such as RNA modifications, have also attracted considerable attention. *N*^6^-methyladenosine (m^6^A), the most abundant RNA modification in eukaryotic cells, affects various biological functions and diseases [123], and it has been reported to regulate myeloid cell activation via MASLD. Specifically, DNA damage-inducible transcript 4 (DDIT4) is highly important for regulating cell growth and survival, particularly under various stress conditions, and its transcripts are m^6^A-decorated to decrease its mRNA stability, leading to reduced protein levels of DDIT4, whereas the induction of DDIT4 can mitigate MASLD by reducing macrophage effector function [124]. In general, research on m^6^A in immune cells at the onset and advancement of MASLD is currently emerging at a fast pace.

### Inflammatory signaling pathways in immune cells

Notch signaling is a highly conserved pathway that serves as the primary juxtacrine signaling pathway used for direct cell-to-cell communication between adjacent cells, influencing numerous cell fate decisions in the development of multicellular organisms [125]. The two main players in the Notch pathway are the Notch receptors and the Notch ligands of the Delta/Serrat/Lag-2 (DSL) family. Canonically, cell membrane-tethered Notch ligands bind to the Notch receptor on neighboring cells, leading to proteolytic cleavage of the Notch receptor and release of the Notch intracellular domain (NICD), which subsequently translocates to the cell nucleus, where it complexes with the CSL-type DNA binding proteins CBF1 (RBPJ), Su(H), and Lag-1 to regulate gene expression [126]. Recently, RBPJ was reported to play an essential role in controlling monocyte-derived macrophage differentiation during MASH development in mice, as *Rbpj* deficiency in monocytes blunted inflammatory macrophage and monocyte-derived Kupffer cell differentiation. Mechanistically, *Rbpj* deficiency in macrophages promoted lipid uptake driven by elevated CD36 expression in Ly6C^lo^ monocytes, enhancing their protective interactions with endothelial cells [127]. These findings reveal a crucial role for the Notch-RBPJ signaling pathway in controlling the differentiation, localization, and function of liver macrophage subsets in MASH. Similarly, another interesting study revealed that *Rbpj* deficiency in myeloid cells decreased experimental MASH by increasing the expression of genes related to fatty acid degradation and peroxisomal fatty acid oxidation [128], suggesting that targeting Notch signaling in macrophages may constitute a novel therapeutic strategy for MASH treatment. Interestingly, Notch1 activation in macrophages increased the levels of exosomal miR-142a-3p, impeding Treg differentiation by targeting transforming growth factor beta receptor 1 (TGFβR1) on T cells. Furthermore, Treg depletion in macrophage-specific Notch1-deficient mice significantly abolished the ameliorative effect of macrophage Notch 1 deletion on hepatic steatosis during MASLD development [78], highlighting the crucial role of the Notch1/exosomal miR-142a-3p/TGFβR1 pathway in macrophages by regulating Treg differentiation during MASLD progression.

The cyclic GMP‒AMP synthase (cGAS)-stimulator of interferon genes (STING) pathway, a key mediator of inflammation, has emerged as a critical mechanism for sensing and regulating the cellular response toward microbial and host-derived DNAs [129]. Recently, mitochondrial DNA (mtDNA) has been recognized as an endogenous damage-associated molecular pattern (DAMP), thus activating the innate immune response and promoting inflammation after release into the cytosol and circulation [130]. Kupffer cells are among the first cells to respond to hepatocyte damage; they act as scavengers and phagocytes in the liver. Strikingly, mtDNA from lipotoxicity-induced hepatocytes induced TNF-α and IL-6 expression in Kupffer cells, whereas STING deficiency attenuated this inflammation, suggesting that STING functions as a mtDNA sensor in Kupffer cells, contributing to MASH progression [131]. In addition, mice with disruption of STING in macrophages exhibited less hepatic steatosis, inflammation and fibrosis after high-fat diet or MASH diet feeding, suggesting that the STING pathway may be a therapeutic target for MASLD [132]. In addition to mtDNA, high-fat diet-induced oxidative stress was able to activate the STING pathway in liver macrophages. Myeloid cell-specific STING knockout increased nuclear yes-associated protein (YAP) activity, reduced lipid accumulation, and increased the expression of autophagy-related proteins, including ATG5, ATG7, and light chain 3B, but decreased lipid droplet protein perilipin2 expression, highlighting the regulatory mechanism of STING-driven YAP activity on lipid control in MASLD [133]. Despite many relevant studies on the functional role of the STING pathway in macrophages during MASLD, the STING pathway may be relevant in other cells, such as hepatocytes, neutrophils and hepatic stellate cells, which needs to be further investigated.

The “don’t-eat-me” signal cluster of differentiation 47 (CD47), a transmembrane glycoprotein expressed on the surface of various cell types, prevents the inadvertent phagocytosis of live cells owing to CD47-mediated activation of the signal-regulatory protein α (SIRPα) receptor on myeloid cells [134]. The CD47-SIRPα axis plays a pivotal role in physiological tissue homeostasis and is a promising therapeutic target in cancer immunotherapy [135]. Intriguingly, the accumulation of necroptotic hepatocytes in human and mouse MASH livers is closely related to the upregulation of CD47 on necroptotic hepatocytes and SIRPα on liver macrophages. Importantly, treatment with either anti-CD47 or anti-SIRPα antibodies ameliorated liver inflammation and fibrosis by enhancing the clearance of necroptotic hepatocytes, suggesting that therapeutic blockade of the CD47-SIRPα axis acts as a strategy to dampen MASLD progression [136]. Another study confirmed that anti-CD47 antibody treatment did not affect mouse body weight, fat mass or liver steatosis but markedly reduced liver immune cell infiltration and fibrosis [137]. Since necroptotic hepatocyte-released DAMPs contribute to liver inflammation in MASH, we can speculate that the combined use of anti-CD47 or anti-SIRPα and potential drugs for correcting metabolic disorders may systematically mitigate MASH (Fig. 4).

### Hepatocyte-released signals orchestrate immune cell infiltration

Building on the intricate interplay between immune cell recruitment and liver inflammation, hepatocyte-derived signals further orchestrate immune infiltration in MASLD, amplifying disease progression through diverse molecular mechanisms. As MASLD progresses, metabolic disturbances such as excessive triglyceride storage, de novo lipogenesis, lipotoxicity, ER stress, mitochondrial dysfunction, and oxidative stress culminate in hepatocyte injury, leading to apoptosis, necrosis, and necroptosis and the release of damage-associated molecular patterns (DAMPs) and proinflammatory mediators that drive immune cell infiltration and liver inflammation [138]. Furthermore, hepatocyte senescence exacerbates this inflammatory cascade through the senescence-associated secretory phenotype (SASP) [139].

Hepatocytes actively contribute to immune cell recruitment through various mechanisms, perpetuating inflammation and disease progression. Hepatocyte-derived extracellular vesicles (EVs) facilitate monocyte adhesion via integrin β1 (ITGβ1)-dependent pathways [140], whereas IRE1A activation in hepatocytes induces the release of ceramide-enriched EVs, which further recruit monocyte-derived macrophages and exacerbate liver inflammation [141]. Given their central role in controlling liver inflammation, EVs represent potential therapeutic targets for mitigating liver injury and immune-driven disease progression. The overexpression of adenosine kinase (ADK) in hepatocytes contributes to excessive fat accumulation and mitochondrial dysfunction, leading to the release of mitochondrial DNA (mtDNA) and lipids, which serve as danger signals. These signals activate macrophages through the STING pathway, leading to the upregulation of the expression of proinflammatory cytokines (IL-1β, IL-6, IL-10, and TNF-α) and increased expression of Ly6c2, a key marker of inflammatory monocyte recruitment [142]. In response to metabolic stress, hepatocytes secrete NKG2D ligands through NKG2D signaling, promoting IL-17A expression in hepatic γδT cells. IL-17A then induces hepatocytes to release chemokines, which recruit proinflammatory myeloid cells into the liver [86]. Under stress conditions in both human and mouse fibrosis, hepatocytes secrete IL-33, which drives the accumulation of group 2 innate lymphoid cells (ILC2s). Moreover, in high-fat, high-fructose diet (HFFD)-fed mice and patients with MASH, infiltrating immune cells secrete acetylcholine, which activates CHRNA4, a calcium channel receptor in hepatocytes. CHRNA4 activation induces calcium influx and triggers NF-κB and MAPK signaling cascades, leading to the secretion of TNF-α, CCL2, and CCL5, which further amplify immune cell infiltration and liver inflammation [143]. Notably, CHRNA4 inhibition by lobeline prevents acetylcholine binding, thereby reducing immune cell recruitment and mitigating MASH progression [143].

As MASLD progresses, chemokines orchestrate the recruitment of circulating immune cells to sites of injury. Among the earliest responders are monocytes, which infiltrate the liver primarily via CCL2 signaling [144]. Once they reach the inflammatory site, neutrophils further increase immune activation by recruiting monocytes via LL-37, azurocidin, cathepsin G (CTSG), human neutrophil peptides 1--3 (HNP1--3) and proteinase 3 (PR3) [145]. Additionally, lymphocyte recruitment is mediated by distinct chemokine axes, including the CXCR6‒CXCL16 pathway for natural killer T (NKT) cells, the CXCR3‒CXCL9‒CXCL10 pathway for inflammatory hepatic CXCR3^+^Th17 (ihTh17) cells, and the CCR2‒CCR5 signaling pathway for γδT cells [74, 144, 146, 147].

Taken together, these findings underscore the critical role of hepatocyte-derived signals in orchestrating immune cell infiltration during MASLD, highlighting potential therapeutic targets to disrupt this inflammatory crosstalk and alleviate disease progression.

## Dysfunction of extrahepatic organs contributes to inflammation in MASLD

### Adipose tissue

Numerous studies on obesity, MASLD, and insulin resistance have emphasized the critical role of adipose tissue macrophages (ATMs) in driving inflammatory processes and metabolic dysregulation. These studies highlighted the increased inflammatory properties of ATMs and their accumulation in adipose tissue as key contributors to systemic insulin resistance and metabolic liver disease. For example, seminal work has demonstrated that obesity is associated with marked infiltration of proinflammatory macrophages into adipose tissue, which is correlated with insulin resistance and low-grade systemic inflammation [148, 149]. More specifically, the phenotypic shift of macrophages in adipose tissue has been linked to metabolic impairment. CD11c^+^CD206^+^ macrophages, a proinflammatory subset, have been shown to be directly associated with insulin resistance [150]. This macrophage phenotype promotes the secretion of cytokines and inflammatory mediators that interfere with insulin signaling pathways, exacerbating metabolic dysfunction. Studies from experimental models have provided additional mechanistic insights. For example, high-fat diet-induced ATM activation triggers the upregulation of proinflammatory gene expression in mouse models [151]. Importantly, this inflammatory response precedes the onset of liver inflammation, suggesting that adipose tissue inflammation is an initiating factor that propagates to other organs, including the liver. The causative role of ATMs in systemic metabolic derangements is further supported by studies showing that genetic or pharmacological ablation of ATMs normalizes whole-body insulin sensitivity [152]. This finding underscores the pivotal role of ATMs in linking adipose tissue inflammation to systemic insulin resistance and metabolic disease progression. Collectively, these studies establish ATMs as central players in the inflammatory cascade that underpins the metabolic dysfunction observed in obesity and MASLD. Their accumulation, activation, and phenotypic shift not only disrupt adipose tissue homeostasis but also contribute to downstream effects on liver metabolism and systemic insulin sensitivity. Clinical studies have further elucidated the role of adipose tissue inflammation in the progression from MASL to MASH, emphasizing its systemic impact on metabolic and liver pathophysiology. Research on adipose tissue obtained from bariatric surgery patients demonstrated an upregulation of inflammatory gene expression as patients transitioned from MASL to MASH. This progression was accompanied by a shift in macrophage phenotypes, with a notable increase in the frequency of proinflammatory CD11c^+^CD206^+^ and CD11c^+^CCR2^+^ macrophages. These macrophages produce elevated levels of cytokines in culture, establishing a clear link between adipose tissue inflammation and advanced liver disease [153].

More recently, single-cell sequencing has provided a deeper understanding of the cellular and molecular landscape of adipose tissue in obesity. Investigating cell‒cell ligand‒receptor interactions and obesity-enriched signaling pathways in white adipose tissue (WAT) [154] highlighted a dramatic shift from immunoregulatory mechanisms in lean WAT to the establishment of inflammatory networks in obese WAT. This switch disrupts tissue homeostasis and fosters a proinflammatory microenvironment that exacerbates systemic metabolic dysfunction. Building on this foundation, a recent study explored the role of unique vascular macrophage subsets within adipose tissue, analyzing samples obtained from bariatric patients stratified by liver histology [155]. This study identified a distinct subset of vascular macrophages critical for maintaining adipose tissue vascular integrity, including resident vasculature-associated macrophages (ResVAMs) and distinct metabolically active macrophages (MMacs) [155]. ResVAMs expressed high levels of *LYVE1* and *FOLR2*, in addition to a set of conserved markers reminiscent of resident macrophages. MMacs, on the other hand, expressed elevated levels of *TREM2*, *GPNMB*, *MARCO* and *TIMD4* and were exclusively localized around blood vessels. Importantly, during MASH progression, ResVAMs but not MMacs are effectively replenished by a monocyte-derived transitional macrophage subtype. MMacs exhibit unique immunomodulatory and reparative properties, which are progressively lost as patients progress toward MASH. This loss coincided with the breakdown of vascular integrity in adipose tissue, a key event linked to the worsening of metabolic dysfunction and liver disease [155]. Taken together, the current data suggest that the loss.

Vascular integrity in adipose tissue represents a pivotal event that is closely associated with the exacerbation of metabolic dysfunction and liver disease. This phenomenon mirrors the critical role of the gut vascular barrier, where the integrity of this barrier is crucial for preventing systemic inflammation and maintaining metabolic homeostasis [156, 157]. In adipose tissue, a similar vascular barrier exists, which is also safeguarded by specialized macrophages. These macrophages play a key role in regulating the permeability of the barrier and thus in controlling the inflammatory processes that can lead to disease states [155]. Together, these findings underscore the complex interplay between adipose tissue inflammation, macrophage phenotypes, and metabolic progression from MASL to MASH. These findings emphasize the importance of both the cellular composition and the functional status of adipose tissue immune cells in determining systemic and hepatic outcomes. These insights suggest that targeting adipose tissue inflammation and preserving vascular macrophage subsets could represent promising therapeutic strategies for interrupting the progression of MASLD. Notably, a recent study revealed that uncoupling protein 1 (UCP1), which is exclusively expressed in brown and beige adipocytes, governs liver extracellular succinate. The deficiency of UCP1 led to elevated extracellular succinate in liver tissue, thereby driving inflammation via succinate receptor 1 (SUCNR1) in liver-resident stellate cells and macrophage populations [158]. This study further highlights the crosstalk between adipose tissue and hepatic inflammation in the context of MASLD.

Growing evidence suggests that obesity-related expansion of adipose tissue is associated with infiltration and activation of immune cells, especially adipose tissue macrophages (ATMs), which also contribute to MASLD progression since their proinflammatory cytokine repertoire leads to the adipose tissue secretome [159]. Although numerous studies have highlighted the importance of liver macrophage heterogeneity in MASLD, the functions of ATM subsets in adipose tissue are not fully understood. Growth differentiation factor 15 (GDF15) is a cytokine belonging to the transforming growth factor-β superfamily. Although GDF15 is a promising target for obesity [160, 161], the function and cellular source of GDF15 during MASLD are not fully understood. Interestingly, biopsies from well-characterized patients with defined obesity, type 2 diabetes (T2D) and MASLD status, along with male mouse models of obesity and MASLD, revealed that adipose tissue is the primary source of GDF15 during the early stages of obesity and T2D, where GDF15 expression is increased through the accumulation of macrophages, and inactivation of *Gdf15* in macrophages exacerbates obesity in mice [162]. However, as the disease progresses toward MASH, *Gdf15* expression is induced in the liver, and hepatocytes, rather than macrophages, become its primary source [162]. These findings highlight the complexity of GDF15 regulation in metabolic diseases and identify potential therapeutic approaches to increase endogenous GDF15 levels. Importantly, in addition to macrophages, cytotoxic T cells in visceral adipose tissue (VAT) are considered critical regulators of MASH pathogenesis, which links adipose tissue inflammation to liver disease [163].

### Gut

The complicated progression of MASLD extends beyond the liver, driven by the “gut‒liver axis”, where diet and genetic and gut‒liver interactions affect disease outcomes [164]. A growing body of evidence suggests that gut bacterial translocation plays an essential role in the development of MASLD. Recently, intestinal IL-33 promoted microbiota-derived trimethylamine *N*-oxide synthesis and accelerated MASLD progression, whereas global or intestinal deletion of *Il33* in mice ameliorated metabolic disorders, inflammation, and fibrosis associated with MASLD [165]. Additionally, H_2_S produced by the gut microbiota triggers the autophagic death of hepatic c-kit^+^ cDC1s, thereby initiating a liver inflammatory cascade in MASH [166]. Targeting the gut‒liver axis, especially by preventing bacterial translocation, can effectively modulate the progression of MASLD. Oat beta-glucan, a nondigestible polysaccharide, was shown to decrease hepatic leukocyte infiltration, especially that of MoMFs, to dampen fibrosis development in diet-induced MASLD, which was associated with the reversal of diet-induced unfavorable changes in microbiota composition and a subsequent reduction in the translocation of TLR4 ligands [167]. In addition, naringenin cationic lipid-modified nanoparticles (NP-NAR) have been reported to alleviate MASLD progression by promoting fatty acid oxidation and modulating the gut microbiota, leading to the amelioration of lipid dysbiosis, oxidative stress, insulin resistance, and inflammation [168]. Moreover, the gut microbiota is involved in the protective effects of time-restricted feeding (TRF) in MASLD via the production of 2-hydroxy-4-methylpentanoic acid (HMP), and oral supplementation with HMP alleviated inflammation and fibrosis in a MASH model [169]. Taken together, these studies suggest promising prospects for targeting the gut‒liver axis to treat hepatic inflammation in MASLD.

In the past decade, many studies have focused on the underlying mechanisms of bile acid metabolism, gut metabolites, and the gut microbiome in the development and progression of MASLD. Fewer studies have investigated the functional role of gut immunity in MASLD. A recent study demonstrated that activated intestinal B cells were increased in mouse and human MASH samples and promoted metabolic T-cell activation in the gastrointestinal tract independent of TCR signaling. More interestingly, this process is not dependent on the gut microbiota. Furthermore, genetic or therapeutic depletion of systemic or gastrointestinal B cells ameliorated T-cell-driven inflammation and fibrosis in MASH by inhibiting the IgA-FcRγ signaling axis on hepatic macrophages [170]. Additionally, another study revealed that gut inflammation exacerbates hepatic steatosis caused by a HFD through the inhibition of hepatic VLDL-TG secretion [171]. Therefore, gut inflammation may directly contribute to the progression of MASLD, and gut immunity plays a crucial role in its pathogenesis.

## Immunoregulatory therapies for MASLD

**Fig. 5:**
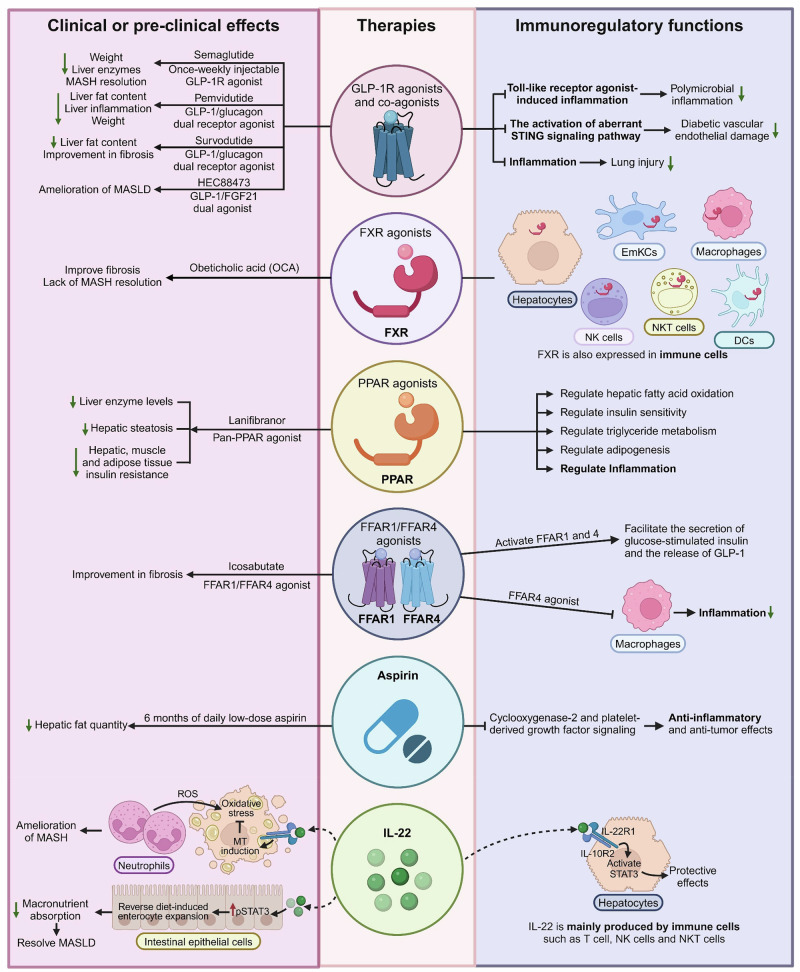
Immunoregulatory therapies for MASLD. Although most pharmacological strategies primarily target metabolic processes, aiming at ameliorating metabolic dysfunction-driven stress and injury in hepatocytes, these potential “MASH therapies” also inhibit the immune cell-mediated inflammatory response

The new nomenclature MASLD emphasizes the essential role of metabolic dysfunction in driving the pathogenesis and outcomes of the disease. Therefore, most pharmacological strategies primarily target metabolic processes, aiming at ameliorating metabolic dysfunction-driven stress and injury in hepatocytes. Very few drugs in development for MASH have direct anti-inflammatory or antifibrotic effects [172]. For example, the first FDA-approved drug is an oral, liver-directed thyroid hormone receptor beta-selective agonist that plays a key role in regulating lipid metabolism, cholesterol synthesis, and fatty acid oxidation [173, 174]. Another example of a “metabolic drug” is denifanstat, an oral fatty acid synthase (FASN) inhibitor, which led to statistically significant improvements in disease activity, MASH resolution, and fibrosis in a multicenter, double-blind, randomized, placebo-controlled phase 2b trial [175]. To date, the efficacy of agents that directly target immune cell function and recruitment is still lacking, since the dual CCR2/CCR5 inhibitor cenicriviroc did not demonstrate antifibrotic efficacy after 1 year of treatment in MASH patients with liver fibrosis in a phase III trial [176]. These metabolism regulation-based therapies not only correct metabolic disorders but also may inhibit the immune cell-mediated inflammatory response. Here, we review the impact of several potential “MASH therapies” regarding their effects on immune mechanisms (Fig. 5).

### GLP-1R agonists and coagonists

The hormone glucagon-like peptide (GLP) plays an integral role in regulating blood glucose levels, lipid metabolism, and several other critical biological functions by interacting with the GLP-1 receptor (GLP-1R), which is a vital component of the G protein-coupled receptor (GPCR) family and is expressed primarily on the surfaces of various cell types [177]. Recently, GLP-1 receptor (GLP-1R) and its agonists have garnered widespread attention in the medical community because they are highly potent treatments for obesity and type 2 diabetes [178]. Although GLP-1Rs are not expressed on hepatocytes [179], GLP-1R agonists have been demonstrated to indirectly influence the liver via their effects on insulin resistance, blood glucose levels and body weight. In addition to these improvements in metabolic disorders, GLP-1 signaling also plays an important role in suppressing inflammation. For example, central GLP-1R activation inhibits Toll-like receptor agonist-induced inflammation, thus reducing the severity of polymicrobial inflammation [180]. GLP-1R agonists protect against diabetic retinal vasculature and inflammation through prohibiting the activation of aberrant STING signaling pathways [181]. The anti-inflammatory effects of GLP-1 in lung injury have also been summarized elsewhere [182]. These studies suggest that GLP-1R agonists may play a beneficial role in inhibiting inflammation during MASLD.

Many clinical trials have assessed the use of GLP-1R agonists for obesity and type 2 diabetes [183]. Given the overlap of MASLD with obesity and diabetes, GLP-1R agonists have been recognized as potential treatment options for MASH. For example, semaglutide, a once-weekly injectable GLP-1R agonist, resulted in significant weight loss, histological resolution of steatohepatitis and improvements in cardiovascular biomarkers [184], although it did not meet the histological antifibrotic effect threshold or reverse cirrhosis in a separate study [185]. Semaglutide is currently undergoing phase III trials for MASLD with fibrosis and compensated MASH cirrhosis; interim results show a significant improvement in fibrosis for the noncirrhotic population at 72 weeks [186]. In addition, dual and triple incretin agonists that combine GLP-1 with glucagon or GIP (glucose-dependent insulinotropic polypeptide) activity to enhance metabolic effects are being developed [187]. Recently, a 12-week randomized, double-blind, placebo-controlled clinical trial (NCT05006885) revealed that pemvidutide, a GLP-1/glucagon dual receptor agonist, may be an effective treatment for MASH, as pemvidutide treatment significantly reduced liver fat content, noninvasive biomarkers of liver inflammation, and body weight compared with placebo [188]. Survodutide, a GLP-1/glucagon dual agonist, resulted in significant weight loss and histological MASH resolution as well as fibrosis improvement after one year of treatment [189]. Although the additional benefits of stimulating glucagon in conjunction with GLP-1 for resolving inflammation are remarkable, the mechanisms (e.g., potential effects on immune cells) are currently incompletely understood.

Fibroblast growth factor 21 (FGF21) is an endogenous liver-secreted hormone that has therapeutic potential for treating obesity, type 2 diabetes, and MASLD [190]. In a randomized, double-blind, placebo-controlled phase Ib/IIa study (NCT05943886), the GLP-1/FGF21 dual agonist HEC88473 was generally safe and well tolerated in patients with MASLD. Five-week treatment significantly reduced the hepatic fat fraction and improved glycemic control and lipid profiles [191]. At present, several FGF21 agonists are in development as monotherapies for MASH [50]. Although GLP-1R agonists are not yet approved solely for treating MASH, major society guidelines and consensus statements have incorporated GLP-1R agonists in treatment pathways [10, 183].

### FXR agonists

Farnesoid X receptor (FXR), a bile acid sensor highly expressed in the liver and intestine, can regulate the expression of genes involved in the pathogenesis of MASH by regulating cholesterol and bile acid homeostasis, hepatic gluconeogenesis, lipogenesis, intestinal barrier integrity, bacterial translocation and the gut microbiota [192, 193]. FXR, like other bile acid receptors, is also expressed in various innate immune cells, such as monocytes and macrophages, dendritic cells, and NK and NKT cells, thus modulating several aspects of innate immunity [194]. FXR has been proposed as a pharmacological target in MASH, since FXR controls multiple pathogenetic pathways relevant to MASH. Although the immunoregulatory role of FXR in MASLD has not been clarified, myeloid cells have been reported to mediate the hepatoprotective effects of pharmacological FXR agonists in sclerosing cholangitis [195]. In the past decade, numerous FXR agonists have been assessed for the treatment of MASH, although side effects such as itching or increases in low-density lipoprotein cholesterol are frequently dose limiting, as summarized in a previous review [192]. The most prominent example of a pharmacological FXR agonist is obeticholic acid (OCA). Despite positive antifibrotic results for OCA in the phase 3 REGENERATE trial [196], the lack of MASH resolution and safety profile led to the definite termination of the OCA development program for MASH.

### PPAR agonists

PPARs are fatty acid sensors that regulate hepatic fatty acid oxidation, insulin sensitivity, triglyceride metabolism, adipogenesis and the inflammatory response [197, 198]. Accumulating evidence shows that the PPAR family plays a vital role in controlling the diverse immune functions of macrophages. For example, PPARγ and PPAR-β/δ are involved in stimulating M2 macrophage polarization [199]. Lanifibranor is a pan-PPAR agonist that modulates key metabolic, inflammatory, and fibrogenic pathways in the pathogenesis of experimental MASH models [200]. In experimental settings, the concurrent agonism of the different PPAR isoforms PPARα, PPARβ/δ and PPARγ allows the simultaneous targeting of metabolic, inflammatory and fibrogenic pathways in different hepatic cell populations [200], which is more effective than targeting only one (or two) of the PPAR isoforms. In a phase 2b, double-blind, randomized, placebo-controlled trial (NCT03008070) evaluating the efficacy and safety of lanifibranor in MASH patients with severe disease activity, the percentage of patients who had a decrease of at least 2 points in the SAF-A score (the activity part of the Steatosis, Activity, Fibrosis [SAF] scoring system that incorporates scores for ballooning and inflammation) without worsening fibrosis was significantly greater with the 1200-mg once-daily dose of lanifibranor than with the placebo [201]. Another phase 2, single-center randomized trial (NCT03459079) also demonstrated that lanifibranor treatment significantly reduced hepatic steatosis and improved hepatic, muscle and adipose tissue insulin resistance in patients with type 2 diabetes and MASLD [202].

### FFAR1/FFAR4 agonists

Free fatty acid receptor (FFAR) 1 and 4 (also known as GPR40 and GPR120, respectively) are proposed to play crucial roles in a variety of physiological and pathophysiological processes, especially in metabolic disorders. FFAR1 and 4 are activated by medium- and long-chain free fatty acids and are putative targets for the treatment of type 2 diabetes by facilitating the secretion of glucose-stimulated insulin and the release of GLP-1 [203, 204]. In addition, the FFAR4 agonist improved glucose tolerance, increased insulin sensitivity and decreased hepatic steatosis in high-fat diet-fed obese mice. Importantly, the FFAR4 agonist exerted potent anti-inflammatory effects on macrophages in vitro and in obese mice in vivo [205]. Recently, in a phase IIb randomized controlled trial (NCT04052516), 52 weeks of once-daily treatment with icosabutate, an oral, semisynthetic FFAR1/FFAR4 agonist, was well tolerated. In patients with MASH and liver fibrosis (stages F1--F3), there was an apparent beneficial effect of icosabutate on surrogate histological endpoints, particularly fibrosis improvement, although the prespecified primary endpoint of MASH resolution without worsening of fibrosis was not met [206].

### Aspirin

Aspirin is a common drug for relieving minor aches, pains and fevers. Accumulating studies suggest that aspirin exerts anti-inflammatory and antitumor effects by inhibiting cyclooxygenase-2 and platelet-derived growth factor signaling, representing a promising and low-cost strategy for the treatment of MASLD [207–209]. Interestingly, in a phase 2 randomized clinical trial (NCT04031729) of 80 individuals with MASLD, 6 months of daily low-dose aspirin significantly reduced hepatic fat quantity compared with placebo, but the findings are preliminary and require confirmation in a larger population [210].

### Interleukin-22

Interleukin-22 (IL-22) is a unique cytokine that is produced mainly by immune cells such as T cells, NK cells and NKT cells. IL-22 functions as a tissue-protective cytokine during liver injury by activating STAT3 in hepatocytes through a receptor complex composed of IL-22R1 and IL-10R2 [211]. A previous study revealed that IL-22 reversed neutrophil-derived reactive oxygen species and the activation of stress kinases via the induction of metallothionein in hepatocytes, ultimately ameliorating MASH [212]. A recent study also reported that IL-22 resolved MASLD via the entry of IL-22R1 but not hepatic IL-22R1, as demonstrated by the finding that hepatocyte-specific *Il22r1* ablation still responded to IL-22 therapy, resulting in reduced body and liver weights, decreased liver damage, and decreased collagen accumulation [213]. Nonetheless, at present, these results are model- and insult specific. Nonetheless, the hepatoprotective effects of IL-22 in MASLD support further exploration as a therapeutic approach.

## Conclusions and Perspectives

MASLD is a complex, dynamic disease whose steatotic and fibrotic activity waxes and wanes in response to diverse genetic, epigenetic and environmental modifiers. Inflammation in MASLD is rarely linear in its development and progression; rather, it fluctuates between flares and resolution, which may explain why the highly dynamic parameter of inflammation is a weaker prognostic feature than fibrosis when it is captured at a single time point via liver histology [172]. Recent technological advances in biology and immunology, including scRNA-seq, snRNA-seq and spatial transcriptomics, have revolutionized our view of the pathogenesis of MASLD, particularly regarding inflammation, and promise to fill gaps in our knowledge [214]. Although these single-cell techniques reveal the transcriptomic changes occurring in cells and tissues, no single methodology can capture all the key and relevant information. Therefore, a combination of approaches that integrate transcriptomics with genomics, epigenomics, proteomics or metabolomics is essential to fully understand the complexity of MASLD, providing a rich and highly comprehensive view of MASLD development and progression. However, analysis of these multimodal datasets is currently a major area of focus in the field [215]. The application of generative artificial intelligence (AI) may present a transformative opportunity to advance our understanding and management of MASLD, such as tabular medical data analysis, multiomics data analysis, the generation of synthetic patient data, enhancing the medical imaging field for MASLD, and advancing personalized medicine approaches for MASLD [216, 217].

Immunological studies on the pathogenesis of MASLD have revealed the key role of macrophages, whereas neutrophils have received less attention [218]. However, neutrophils are the most abundant leukocyte type in the human circulation [219], and neutrophil infiltration is commonly observed in human MASH, as well as in alcohol-related SLD [220, 221]. Therefore, defining circulating and liver infiltrating neutrophil heterogeneity and function during MASLD may further our understanding. In addition, since the old acronym “NAFLD” has been changed to the new name “MASLD”, a new subgroup of MetALD has emerged. Owing to substantial underreporting and little recognition of the effect of alcohol on SLD, a more accurate prognose and effective management of SLD, addressing both metabolic and alcohol-related factors is crucial to appropriately diagnose, manage, and address the disease [222, 223]. Daily drinking is closely associated with high blood pressure, hypertriglyceridemia, and hyperglycemia, which may suggest a metabolic origin for SLD and lead to underestimation of the critical role of alcohol [223]. The effects of (low) alcohol consumption on MASLD progression and MASH immune pathogenesis are incompletely understood.

Although the first pharmacotherapeutic for MASLD and MASH, resmetirom, has been approved for clinical use [224], we expect more targeted treatments owing to the modest efficacy of resmethrin [2]. Given the complicated pathogenic mechanisms of MASLD, the future therapeutic landscape for MASH should include drugs that target different pathophysiological drivers of the disease. In addition, combination therapies will eventually be used, despite early data from clinical trials being negative [225]. With a better understanding of MASLD heterogeneity, individualized therapy could be recommended by selecting the best agent or the best combination of agents that is matched to the patient profile.
